# The impact of self-control on aggressive behavior: the chained mediating role of hostile attribution bias and positive/negative implicit affect

**DOI:** 10.3389/fpsyg.2025.1638323

**Published:** 2025-10-15

**Authors:** Qiannan Ma, Xiaoyin Wang, Lanxi Liu

**Affiliations:** ^1^Zhoukou Normal University, Student Mental Health Education Center, Zhoukou, China; ^2^Henan University, Faculty of Education, Kaifeng, China

**Keywords:** self-control, aggressive behavior, hostile attribution bias, implicit affect, vocational students

## Abstract

**Objective:**

This study examined the direct impact of self-control on aggressive behavior and the mediating roles of hostile attribution bias and both positive and negative implicit affect.

**Methods:**

A questionnaire survey was conducted among 545 vocational students at a vocational school in Henan Province, utilizing the Self-Control Scale, Hostile Attribution Bias Questionnaire, Implicit Positive–Negative Affect Test, and Aggressiveness Behavior Scale as measurement tools. Data were analyzed using SPSS 26.0 and PROCESS 4.1.

**Results:**

The findings revealed that selfcontrol negatively correlated with aggressive behavior, negative implicit affect, and hostile attribution bias, but showed a positive correlation with positive implicit affect. A significant chained mediating effect of hostile attribution bias and negative implicit affect was observed, while the mediating effect of positive implicit affect was not significant.

**Discussion:**

These results confirm that self-control influences aggressive behavior through a complex psychological pathway, highlighting the critical roles of hostile attribution bias and negative implicit affect in this process. The findings provide a basis for interventions targeting aggressive behavior based on psychological variables.

## 1 Introduction

Vocational students constitute a substantial and distinctive group within China’s educational system (Ma, 2024). By 2023, the number of students enrolled in vocational schools had reached 34.78 million, with 17.38 million attending secondary vocational schools (Yue and Wang, 2025). This large population highlights both the importance of vocational education and the necessity of addressing students’ psychological development and mental health needs in order to promote high-quality educational outcomes. Previous studies have indicated that, due to the tracking mechanism of the education system, secondary vocational students are more likely to experience academic setbacks and career-related confusion, which may foster feelings of inferiority, anger, and hostility. Consequently, their mental health levels tend to be lower than the national adolescent average (Yu and Zhang, 2021). One particularly concerning manifestation is aggressive behavior, defined as intentional physical or psychological harm to others motivated by hostile states (Song et al., 2025). Aggression is relatively common among adolescents, with prevalence rates ranging from 2 to 16%, and a detection rate of 11.4% reported among secondary vocational students (Xiao, 2021). Aggressive behavior has profound negative consequences for both victims and perpetrators. It not only undermines victims’ psychological and physical health but also impairs aggressors’ peer relationships and academic performance (Hu et al., 2023). Moreover, aggression disrupts social development, personality formation, and cognitive functioning (Lin et al., 2024). Thus, examining aggression among secondary vocational students is essential for understanding their personality development (Lawler et al., 2024), while also offering theoretical and practical insights into fostering prosocial behaviors and reducing maladaptive outcomes in this population.

### 1.1 Self-control and aggressive behavior

Factors external to the individual, such as parenting styles, domestic violence, negative peer relationships, and exposure to media violence, are closely associated with the manifestation of aggressive behaviors (Baron and Forde, 2020). Moreover, internal factors such as self-esteem, feelings of relative deprivation, and self-control also play crucial roles in the development of aggressive behaviors (Shi et al., 2017). Notably, self-control is a critical risk factor for the development of aggressive behaviors in children and adolescents (Chester, 2024). Self-control is one of the most powerful abilities of the human mind, conferring significant benefits to individuals. It involves the process of suppressing or overcoming one’s desires and needs to change established or habitual behaviors and ways of thinking, essentially replacing one mode of behavior or thought with another (Tan and Guo, 2008a, 2008b). There are significant individual differences in self-control capabilities; Individuals with higher levels of self-control exhibit stronger adaptability, greater satisfaction in relationships, and overall life satisfaction. Individuals with strong adaptability and high life satisfaction tend to exhibit fewer aggressive behaviors (Buelga et al., 2008). Furthermore, research indicates that individuals with high levels of self-control are able to effectively avoid problematic behaviors such as smoking, drinking, and violence (Narzuki and Basuki, 2022). Thus, we propose hypothesis 1:

*H1:* Self-control negatively predicts aggressive behavior.

### 1.2 The mediating role of hostile attribution bias

Hostile attribution bias (HAB) refers to the cognitive tendency of individuals to perceive others’ motives as intentionally harmful in ambiguous situations (Dodge, 2006). This psychological mechanism is closely related to the level of self-control (Fang et al., 2023). Specifically, the strength of self-control influences how individuals process information and make decisions (Xu T. et al., 2024; Xu X. et al., 2024). Individuals with weaker self-control are less able to rationally analyze others’ behaviors in unexpected situations. Particularly when the situation is unclear, they are more likely to quickly make hostile attributions. The hostile attribution bias model (Dodge, 2006) argues that an individual’s attribution and interpretation of others’ provocative behaviors are the determinants of aggressive behavior. If a person always interprets others’ actions as being hostile, they are more likely to react with aggression (Zhao and Zheng, 2024). Research has shown that hostile attribution bias further strengthens the relationship between self-control and aggressive behavior (Liu et al., 2022). Thus, we propose hypothesis 2:

*H2*: Hostile attribution bias plays a mediating role between self-control and aggressive behavior in adolescents.

### 1.3 The mediating role of positive/negative implicit affect

Affect, as a critical internal factor influencing individuals’ self-control and aggressive behavior (Wu and He, 2011), can be divided into explicit and implicit levels. Unlike explicit affect, which individuals can clearly perceive and directly report, implicit affect refers to unconscious and automatic affective tendencies or states that influence cognition, judgment, and behavior without individuals’ awareness (Hernández et al., 2020). In this study, we define implicit affect as automated positive or negative affective tones that are not consciously processed by individuals. In recent years, increasing attention has been devoted to the role of implicit affect in shaping behavioral decision-making and self-control, particularly in the context of aggressive behavior as a complex social phenomenon. Among these perspectives, cognitive resource theory provides a useful framework for understanding how implicit affect influences self-control and related behaviors (Hou, 2009). This theory suggests that affective states influence individuals’ self-control by affecting the allocation of cognitive resources. Existing studies have shown that individuals who struggle to recognize or regulate negative affect are more likely to display proactive aggressive behaviors (Acland et al., 2024). Moreover, negative implicit affect such as anger, anxiety, or fear is regarded as a persistent drain on cognitive resources (Kappes and Schikowski, 2013). Such depletion not only constrains individuals’ ability for rational analysis and impulse inhibition but may also indirectly increase the likelihood of aggressive behavior by weakening self-control (Dong et al., 2011). In addition, research has found that individuals experiencing negative automatic affective states tend to interpret others’ behavior in a hostile manner, thereby amplifying aggressive impulses (Acland et al., 2024). These findings suggest that negative implicit affect may affect aggressive behavior both by depleting cognitive resources that support self-control and by directly fueling cognitive biases. Thus, we propose hypothesis 3:

*H3:* Negative implicit affect mediates the relationship between self-control and adolescents’ aggressive behavior.

In contrast, positive implicit affect, such as joy and satisfaction, is generally considered to enhance individuals’ efficiency in utilizing cognitive resources, thereby promoting information processing and self-control (Clore and Huntsinger, 2007). From this perspective, positive implicit affect may help buffer impulsive reactions and reduce the likelihood of aggressive behavior. For example, prior research has shown that higher levels of positive affect are associated with stronger self-control, which in turn predicts lower levels of aggressive behavior (Tice et al., 2007). However, empirical findings remain inconsistent. Some studies suggest that the inhibitory effect of positive affect on aggression varies by gender, being significant only among girls (Cristina Richaud and Mesurado, 2016). Other studies have reported that positive implicit affect is not always a protective factor for self-control; in certain contexts, it may actually enhance impulsivity and become a risk factor for aggressive behavior (Salavera et al., 2017). Similarly, Chester (2017) found that positive implicit affect may be positively correlated with aggression. Taken together, although the relationship between positive implicit affect and aggressive behavior remains debated, the overall evidence indicates that positive implicit affect may influence aggressive behavior through its impact on self-control. Thus, we propose hypothesis 4:

*H4:* Positive implicit affect mediates the relationship between self-control and adolescents’ aggressive behavior.

### 1.4 The serial mediating roles of hostile attribution bias and implicit affect

Hostile attribution bias, as a form of negative cognitive distortion, has been shown to directly influence individuals’ emotional experiences, thereby contributing to the occurrence of aggressive behavior (Jiang et al., 2024). Based on the strength model of self-control, adolescents deplete their limited cognitive resources to counteract and regulate their hostile responses (Crusius and Mussweiler, 2012). As cognitive resources are progressively exhausted, adolescents find it increasingly difficult to manage the negative impacts of adverse emotions, leading to a higher likelihood of aggressive behavior (Gutiérrez-Cobo et al., 2018). Furthermore, previous research has confirmed that self-control can inhibit the activation of hostile schemas and reduce hostile attributions and aggressive behaviors by regulating affect and behavior (Shachar et al., 2016). Thus, we propose hypothesis 5:

*H5:* Hostile attribution bias and positive/negative affect serially mediate the relationship between self-control and adolescents’ aggressive behavior.

## 2 Participants and methodology

### 2.1 Participants

This study employed a random sampling method to select 577 vocational students from a vocational school in Henan Province as participants for the questionnaire survey. After excluding invalid questionnaires, a total of 545 valid questionnaires were obtained, resulting in an effective response rate of 94.45%. Of the valid samples, 261 were male (47.9%). The distribution by grade was as follows: 163 students in the first year (29.9%), 122 students in the second year (22.4%), and 260 students in the third year (47.7%). Criteria for invalid questionnaires included extensive missing items, mechanical or patterned responses, and carelessly filled questionnaires, such as contradictory scores on positive and negative items. The study protocol was approved by the Ethics Committee (approval number: 20211010002) and informed consent was obtained from the participants and the school.

### 2.2 Measurement instruments

#### 2.2.1 Self-Control Scale

The Self-Control Scale (Tan and Guo, 2008a, 2008b) was administered to assess participants’ self-control capacity. This 19-item instrument (Cronbach’s *α* = 0.86) comprises five dimensions: impulse control, health habits, resistance to temptation, academic performance, and recreational restraint. Items are rated on a 5-point Likert scale (1 = *strongly disagree*, 5 = *strongly agree*). The total score is calculated by summing all items, with higher scores indicating greater self-control ability.

#### 2.2.2 Hostile Attribution Bias Questionnaire

The Hostile Attribution Bias Questionnaire (Riemann et al., 2013) was used to measure participants’ tendency to attribute hostile intentions to others’ ambiguous behaviors. The questionnaire presents 16 ambiguous scenarios, each followed by both a hostile and a benign interpretation. Participants rapidly evaluate the similarity between each scenario and these interpretations using a 6-point scale (1 = *completely dissimilar*, 6 = *completely similar*). The hostile attribution bias score is calculated as the difference between total hostile attribution scores and total benign attribution scores (Cronbach’s *α* = 0.79), with higher difference scores indicating stronger hostile attribution bias.

#### 2.2.3 Implicit Positive and Negative Affect Test

The Implicit Positive and Negative Affect Test (IPANAT) was used to assess implicit affective tendencies (Hernández et al., 2020). The scale consists of 36 items (Cronbach’s *α* = 0.89) and comprises two dimensions: Implicit Positive Affect (IPA) and Implicit Negative Affect (INA). Participants were presented with six artificial meaningless words (e.g., “*SUKOV*”) as stimuli and were asked to intuitively rate the extent to which each word is associated with three positive affect items (e.g., happy, enthusiastic, energetic) and three negative affect items (e.g., helpless, tense, ashamed). Responses were recorded on a 5-point Likert scale (1 = *not related at all*, 5 = *very strongly related*). IPA and INA scores were calculated by averaging the ratings for positive and negative affect items, respectively. Higher IPA scores indicate stronger implicit positive affect, whereas higher INA scores reflect more pronounced implicit negative affect.

#### 2.2.4 Aggressive Behavior Scale

The Aggressive Behavior Scale (Buss and Perry, 1992) was utilized to measure aggressive tendencies. This 29-item instrument (Cronbach’s *α* = 0.81) consists of four dimensions: physical aggression, verbal aggression, anger, and hostility. All items are rated on a 5-point Likert scale (1 = *strongly disagree*, 5 = *strongly agree*). The total score is obtained by summing all items, with higher scores indicating more pronounced aggressive behavior.

### 2.3 Statistical methods

The statistical analysis for this study was conducted using SPSS 26.0 for data organization and analysis. The analyses included a test for common method bias, descriptive and correlational analyses of the variables, and mediation effect tests. The serial mediation effects of hostile attribution bias and positive/negative implicit affect in the relationship between self-control and aggressive behavior were examined using Model 6 in PROCESS 4.1. This analysis employed a Bootstrap method with 5,000 resamples to estimate the 95% confidence intervals (CIs) for the mediation effects. Mediation was considered statistically significant if the 95% CI did not include zero, and a *p*-value less than 0.05 was considered statistically significant.

## 3 Results

### 3.1 Common method bias

The extent of common method variance was assessed using Harman’s single-factor test. The analysis identified 15 factors with eigenvalues greater than 1, and the variance explained by the first factor was 36.79%, which is below the commonly accepted threshold of 40%. Thus, it can be concluded that common method bias is not a significant concern in this study.

### 3.2 Descriptive statistics and correlation analysis

The results of the descriptive statistics and correlation analysis for each variable are presented in Table 1. Self-control was negatively correlated with aggressive behavior, negative implicit affect, and hostile attribution bias, and it showed a significant positive correlation with positive implicit affect. Aggressive behavior was negatively correlated with positive implicit affect, while negative implicit affect and hostile attribution bias were positively correlated. Positive implicit affect was negatively correlated with both negative implicit affect and hostile attribution bias; conversely, negative implicit affect was positively correlated with hostile attribution bias.

**Table 1:** Descriptive statistics and correlation matrix (*n* = 545).

Variable	Mean ± SD	1	2	3	4	5
1. Self-control	62.41 ± 15.60	1				
2. Aggressive behavior	77.58 ± 21.27	−0.847**	1			
3. Positive implicit affect	32.66 ± 15.60	0.637**	−0.578**	1		
4. Negative implicit affect	22.22 ± 15.43	−0.640**	0.600**	−0.519**	1	
5. Hostile attribution bias	−1.43 ± 18.62	−0.591**	0.562**	−0.544**	0.563**	1

*p < 0.1, **p < 0.05, ***p < 0.01.

### 3.3 Chained mediation model analysis

Following a good model fit, further analysis was conducted using Model 6 to test the mediating mechanisms of hostile attribution bias and positive and negative implicit affect in the relationship between self-control and aggressive behavior. The results indicated that hostile attribution bias and negative implicit affect have both independent and serial mediating effects between self-control and aggressive behavior. However, there was no serial mediation effect involving hostile attribution bias and positive implicit affect, nor was there a simple mediating effect of positive implicit affect between self-control and aggressive behavior.

**Figure 1:**
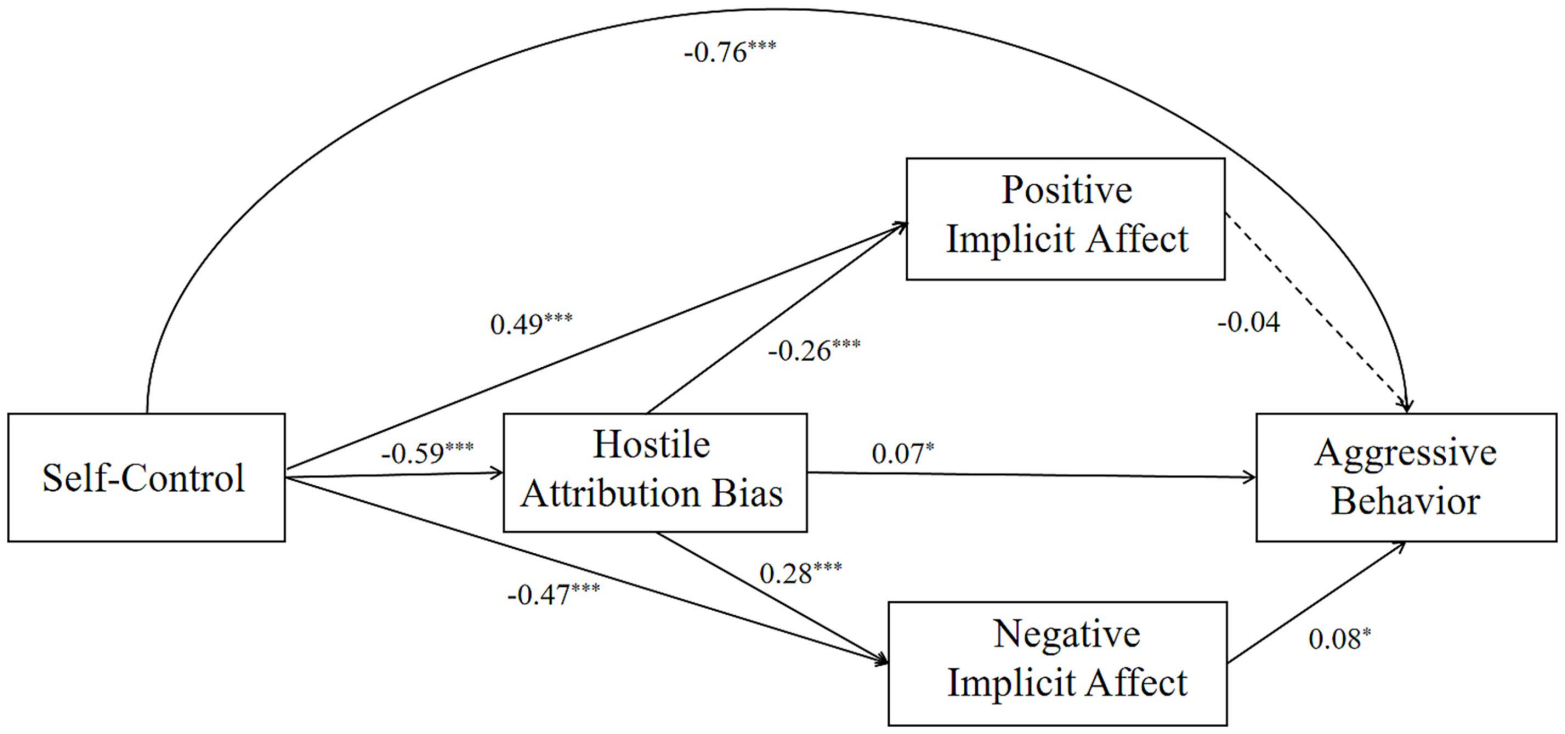
Chained mediation model of self-control predicting aggressive behavior (dashed lines indicate non-significant path coefficients). *p < 0.1, **p < 0.05, ***p < 0.01.

From Table 2, it can be seen that self-control significantly negatively predicts hostile attribution bias (*β* = −0.59, *p* < 0.001), negative implicit affect (*β* = −0.47, *p* < 0.001), and aggressive behavior (*β* = −0.76, *p* < 0.001); It positively predicts positive implicit affect (*β* = 0.49, *p* < 0.001). Hostile attribution bias significantly positively predicts negative implicit affect (*β* = 0.28, *p* < 0.001) and aggressive behavior (*β* = 0.07, *p* < 0.05), and negatively predicts positive implicit affect (*β* = −0.26, *p* < 0.001). Negative implicit affect significantly positively predicts aggressive behavior (*β* = 0.08, *p* < 0.05), while positive implicit affect does not predict aggressive behavior (*β* = −0.04, *p* > 0.05) (See Figure 1).

**Table 2:** Regression analysis of factors influencing aggressive behavior (*n* = 545).

Regression equation		Fit coefficients			R*C	Sig
Dependent variable	Predictor	*R*	*R^2^*	*F*	*β*	*t*
Hostile attribution bias	Self-control	0.59	0.35	309.29	−0.59	−17.59***
Negative implicit affect	Self-control	0.68	0.46	246.57	−0.47	−12.45***
	Hostile attribution bias				0.28	7.46***
Positive implicit affect	Self-control	0.67	0.45	233.56	0.49	12.62***
	Hostile attribution bias				−0.26	−6.68***
Aggressive behavior	Self-control	0.85	0.73	506.54	−0.76	−24.74***
	Hostile attribution bias				0.07	2.56*
	Negative implicit affect				0.08	2.52*
	Positive implicit affect				−0.04	−1.39
Aggressive behavior	Self-control	0.85	0.72	1459.74	−0.85	−38.21***

*p < 0.1, **p < 0.05, ***p < 0.01.

The study conducted a mediation effect test on hostile attribution bias and positive and negative implicit affect using the Bootstrap analysis method. The analysis results are shown in the figure. The standardized estimates for each indirect path and the 95% confidence intervals (CI) for mediation effects are presented in Table 3.

**Table 3:** Translation of bootstrap 95% confidence intervals for indirect effects in different paths.

Path	Effect	95% Confidence Interval		Mediation effect proportion
		Upper limit	Lower limit	
Self-control → hostile attribution bias → aggressive behavior	−0.059	−0.107	−0.011	5.11%
Self-control → negative implicit affect → aggressive behavior	−0.048	−0.094	−0.005	4.16%
Self-control → positive implicit affect → aggressive behavior	−0.027	−0.070	0.018	2.34%
Self-control → hostile attribution bias → negative implicit affect → aggressive behavior	−0.017	−0.036	−0.002	1.47%
Self-control → hostile attribution bias → positive implicit affect → aggressive behavior	−0.009	−0.024	0.005	0.78%
Total indirect effect	−0.124	−0.185	−0.064	10.74%
Direct effect	−1.030	−1.112	−0.949	89.18%
Total effect	−1.155	−1.214	−1.095	

Table 3 indicates that hostile attribution bias and negative implicit affect mediate the relationship between self-control and aggressive behavior, with a total indirect effect of −0.124, accounting for 10.74% of the total effect. Specifically, the following three indirect effects are observed: Indirect effect 1 through the path “self-control → hostile attribution bias → aggressive behavior” yields an effect value of −0.059, constituting 5.11% of the total effect; Indirect effect 2 through the path “self-control → negative implicit affect → aggressive behavior” yields an effect value of −0.048, constituting 4.16% of the total effect; Indirect effect 3 through the path “self-control → hostile attribution bias → negative implicit affect → aggressive behavior” yields an effect value of −0.017, constituting 1.47% of the total effect. The Bootstrap 95% confidence intervals for all three paths do not include zero, indicating that these indirect effects are statistically significant. However, for the paths “self-control → positive implicit affect → aggressive behavior” and “self-control → hostile attribution bias → positive implicit affect → aggressive behavior,” the Bootstrap 95% confidence intervals include zero, suggesting that the serial mediation effects of hostile attribution bias and positive implicit affect are not significant.

## 4 Conclusion and discussion

The present study aimed to investigate the mechanisms through which self-control, hostile attribution bias, and positive/negative implicit affect influence aggressive behavior among Chinese secondary vocational students. Using questionnaire surveys and quantitative analyses, we systematically examined the relationship between self-control and aggressive behavior, and further explored the mediating roles of hostile attribution bias and implicit affect. The results indicated a significant serial mediation effect of hostile attribution bias and negative implicit affect between self-control and aggressive behavior, whereas the mediating role of positive implicit affect was not statistically significant.

### 4.1 The relationship between self-control and aggressive behavior

Self-Control Theory posits that self-control is a critical factor in preventing individual behavioral deviations. Higher levels of self-control help individuals restrain impulses and avoid engaging in aggressive or violent behaviors. Research by Gottfredson and Hirschi suggests that individuals with poor self-control are more likely to make immediate gratification choices that may be detrimental in the long term (Hirschi and Gottfredson, 2000). The results of this study indicate that self-control significantly negatively predicts aggressive behavior. This finding aligns with previous research results (Chester, 2024). Higher levels of self-control may assist individuals in effectively coping with stress and setbacks, thereby avoiding aggressive responses. Additionally, self-control may influence how individuals interpret social interactions, reducing the likelihood of misunderstandings and overreactions.

### 4.2 The mediating mechanism of hostile attribution bias

There is a significant correlation between hostile attribution bias and aggressive behavior, especially among individuals who frequently feel threatened or have experienced multiple conflicts (Van Bockstaele et al., 2020). Although self-control ability typically helps inhibit aggressive behavior, hostile attribution bias may weaken this protective effect. Even individuals with higher levels of self-control may exhibit aggressive responses due to hostile attribution bias. This is because this cognitive bias alters individuals’ fundamental interpretations of social interactions, making them more inclined to perceive hostility in behavior, which may trigger defensive or aggressive reactions, even when such reactions are objectively unnecessary (Subra, 2023). When individuals perceive hostility in others’ behavior, they may resort to aggressive behavior as a form of self-defense, even in situations where it is unwarranted. This occurs because hostile attribution bias directly triggers emotional responses, such as anger or fear, which increase the likelihood of aggressive behavior. Moreover, this bias may also lead to the anticipation of negative outcomes in future social interactions, thereby exacerbating conflicts and hostile behaviors without sufficient justification (Ding et al., 2024).

### 4.3 The chain mediating mechanism of hostile attribution bias and negative implicit affect

As mentioned earlier, hostile attribution bias refers to individuals’ tendency to interpret others’ behavior as hostile, representing a cognitive distortion. Negative implicit affect, on the other hand, refers to negative emotional responses experienced by individuals unconsciously, often triggered by underlying emotional memories or experiences (Lyu et al., 2024). When individuals experience hostile attribution bias, they may misinterpret neutral or ambiguous social cues as hostile, which can trigger negative implicit affect associated with past negative experiences, such as fear, anger, or sadness. These negative implicit affects not only intensify individuals’ emotional responses but may also unconsciously increase their propensity for aggressive behavior (Xu et al., 2023).

Higher levels of self-control typically help individuals interpret others’ behavior in a more objective and peaceful manner, thereby reducing the occurrence of hostile attribution bias. However, even individuals with strong self-control may experience cognitive distortions of hostile attribution under specific pressures or triggering conditions (Kirst et al., 2022). Once hostile attribution bias occurs, it can trigger or exacerbate individuals’ negative implicit affect, which may influence their behavior decisions even when not fully conscious (Ong et al., 2018). The accumulation or triggering of negative implicit affect can prompt individuals to engage in aggressive behavior as a response to perceived threats or injustices, even if such perceptions may be based on erroneous attributions.

### 4.4 The role of negative implicit affect

Negative implicit affect significantly impacts individuals’ cognitive functioning. These affects often require a considerable amount of cognitive resources to process, and when individuals experience intense negative affect, most cognitive resources are redirected to address immediate physiological-response demands. In situations where cognitive resources are heavily depleted, individuals’ ability to process other non-survival-related information becomes limited (Laloyaux et al., 2019). This uneven allocation of resources may lead to biases in information processing, particularly in the interpretation of social situations.

Hostile attribution bias significantly increases the risk of aggressive behavior. When individuals incorrectly interpret others’ actions as hostile, they may engage in aggressive behavior as a defense mechanism to prevent anticipated harm. This reaction occurs in highly emotional and cognitively resource-limited conditions, making it often excessive or inappropriate. This reactive pattern based on erroneous attributions may further exacerbate individuals’ emotional responses, forming a vicious cycle that not only depletes more cognitive resources but may also deteriorate social relationships and lead to more conflicts.

### 4.5 The role of positive implicit affect

The present study found that positive implicit affect did not play a significant role in the chain mediation pathway linking self-control and aggressive behavior. This finding provides novel insights into the relationships among the core variables of the study. First, from the structural perspective of the chain pathway, hostile attribution bias is naturally coupled with negative implicit affect, as its core mechanism involves negative interpretations of others’ behavior that directly elicit anger, anxiety, and other forms of negative implicit affect (Dodge, 2006). In contrast, hostile attribution bias rarely triggers positive automatic affective responses, which makes it difficult for positive implicit affect to exert a significant influence within this pathway. Thus, hostile attribution bias may not be sufficient to activate positive implicit affect, resulting in the disruption of the mediating effect. Second, from a theoretical standpoint, the affect-as-information theory suggests that positive affect tends to foster cognitive flexibility and creative thinking (Bridekirk et al., 2016), thereby enhancing self-control through cognitive broadening and resource integration. However, such effects are more likely to emerge in open-ended tasks, creative contexts, or positive social interactions rather than in conflict-laden contexts dominated by hostile attribution. Within the framework of the present study, the regulatory mechanisms of positive affect may therefore not have been effectively activated.

Moreover, prior research on the relationship between positive affect and aggressive behavior has produced complex and context-dependent findings rather than a uniform conclusion. On the one hand, some studies support the protective role of positive affect. For example, research has found that positive affect can reduce aggressive behavior by enhancing self-control, which is consistent with the broaden-and-build theory, suggesting that positive affect expands individuals’ thought–action repertoires and resources (Tice et al., 2007). On the other hand, other studies highlight the nuanced nature and boundary conditions of this effect. For instance, the inhibitory influence of positive affect on aggression may vary by gender, being significant only among females (Cristina Richaud and Mesurado, 2016). Furthermore, under certain conditions, positive affect may even increase the risk of aggression. Research has indicated that positive affect can be associated with higher impulsivity (Salavera et al., 2017) or, when combined with retaliatory motives, may strengthen rather than inhibit aggressive behavior (Chester, 2017). These findings suggest that positive affect does not universally function as a protective factor but is highly dependent on contextual factors (e.g., cooperation vs. conflict), individual characteristics (e.g., gender, personality traits), and motivational dynamics. Consequently, the influence of positive affect on aggressive behavior appears to be multifaceted, encompassing both inhibitory and facilitative effects that need to be understood within specific psychological and social contexts.

In sum, the nonsignificant role of positive implicit affect identified in this study does not imply its complete ineffectiveness but rather reveals its limited function under the boundary conditions of a hostile attribution-driven chain model. Future research may further investigate the role of positive affect in prosocial contexts, cooperative tasks, or emotion regulation processes to more precisely delineate its mechanisms and scope of influence in different domains of social behavior.

## 5 Limitations and implications

Several limitations should be considered when interpreting the findings of this study. First, the cross-sectional survey design, while capable of revealing correlational relationships and potential mediation mechanisms, precludes definitive causal inferences regarding the relationships between self-control, hostile attribution bias, implicit affect, and aggressive behavior. Future research should employ longitudinal designs (Yu et al., 2022) or experimental paradigms (Acland et al., 2024) to further validate the causal roles of these variables. Second, the data were primarily collected through self-report measures, which may be susceptible to social desirability bias and other self-perception distortions. Although the inclusion of implicit affect measures aimed to mitigate some subjective biases, utilizing multi-method assessments and gathering data from multiple informants would enhance the validity and robustness of the findings. Third, the generalizability of the results may be limited, as the sample consisted exclusively of Chinese vocational school students. Individuals from different educational backgrounds, cultural environments, or age groups may exhibit distinct patterns of aggressive behavior and underlying implicit affective mechanisms. Future studies should extend this research to general high school students, university populations, and other cultural contexts to examine the cross-population applicability of the proposed model. Furthermore, the non-significant role of positive implicit affect in the chained mediation pathway suggests that the regulatory function of positive affect may be context-dependent or subject to specific boundary conditions. Subsequent investigations could explore the potential role of positive affect in cooperative settings, emotion regulation training, or prosocial behaviors to enrich our understanding of the psychological mechanisms underlying aggression. In conclusion, this study provides empirical support for understanding the mechanisms through which self-control, cognitive bias, and implicit affect influence aggressive behavior in adolescents. It also offers a foundation for future longitudinal research, multi-source data collection, and the development of affective intervention practices.

## Data Availability

The datasets presented in this study can be found in online repositories. The names of the repository/repositories and accession number(s) can be found in the article/Supplementary material.

## References

[ref1] Acland E. L. Peplak J. Suri A. Malti T. (2024). Emotion recognition links to reactive and proactive aggression across childhood: a multi-study design. Dev. Psychopathol. 36, 1122–1133. doi: 10.1017/S0954579423000342, PMID: 37039136

[ref2] Baron S. W. Forde D. R. (2020). Childhood trauma, criminogenic social schemas, and violent crime. Deviant Behav. 41, 991–1004. doi: 10.1080/01639625.2019.1596534

[ref3] Bridekirk J. Turcotte J. Oddson B. (2016). Harmonious passions support cognitive resources. Motiv. Emot. 40, 646–654. doi: 10.1007/s11031-016-9561-y

[ref4] Buelga S. Musitu G. Murgui S. Pons J. (2008). Reputation, loneliness, satisfaction with life and aggressive behavior in adolescence. Span. J. Psychol. 11, 192–200. doi: 10.1017/S1138741600004236 18630660

[ref5] Buss A. H. Perry M. (1992). The aggression questionnaire. J. Pers. Soc. Psychol. 63, 452–459. 1403624 10.1037//0022-3514.63.3.452

[ref6] Chester D. S. (2017). The role of positive affect in aggression. Curr. Dir. Psychol. Sci. 26, 366–370. doi: 10.1177/0963721417700457

[ref7] Chester D. S. (2024). Aggression as successful self-control. Soc. Personal. Psychol. Compass 18:e12832. doi: 10.1111/spc3.12832

[ref8] Clore G. L. Huntsinger J. R. (2007). How emotions inform judgment and regulate thought. Trends Cogn. Sci. 11, 393–399. doi: 10.1016/j.tics.2007.08.005, PMID: 17698405 PMC2483304

[ref9] Cristina Richaud M. Mesurado B. (2016). Las Emociones Positivas Y La Empatía Como Promotores De Las Conductas Prosociales E Inhibidores De Las Conductas Agresivas. Acción Psicol. 13, 31–41. doi: 10.5944/ap.13.2.17808

[ref10] Crusius J. Mussweiler T. (2012). When people want what others have: the impulsive side of envious desire. Emotion 12, 142–153. doi: 10.1037/a0023523, PMID: 21604867

[ref11] Ding R. Wang S. Liu J. He W. Pan J. (2024). Maternal supportive responses to adolescents' negative emotions serve as protective factors for adolescents' hostile attribution bias longitudinally. Fam. Process 63, 1531–1550. doi: 10.1111/famp.12946, PMID: 37915232

[ref12] Dodge K. A. (2006). Translational science in action: hostile attributional style and the development of aggressive behavior problems. Dev. Psychopathol. 18, 791–814. doi: 10.1017/S0954579406060391 17152401 PMC2745254

[ref13] Dong G. Zhou H. Zhao X. Lu Q. (2011). Early negativity bias occurring prior to experiencing of emotion. J. Psychophysiol. doi: 10.1027/0269-8803/a000027

[ref14] Fang X. Zhang K. Chen J. Chen M. Wang Y. Zhong J. (2023). The effects of covert narcissism on Chinese college students cyberbullying: the mediation of hostile attribution bias and the moderation of self-control. Psychol. Res. Behav. Manage. 16, 2353–2366. doi: 10.2147/PRBM.S416902 PMC10314775 37396405

[ref15] Gutiérrez-Cobo M. J. Megías A. Gómez-Leal R. Cabello R. Fernández-Berrocal P. (2018). The role of emotional intelligence and negative affect as protective and risk factors of aggressive behavior: a moderated mediation model. Aggress. Behav. 44, 638–646. doi: 10.1002/ab.21788, PMID: 30136277

[ref16] Hernández G. P. Edo S. Quirin M. Rovira T. (2020). A brief version of the implicit positive and negative affect test (IPANAT-18). Psychol. Belg. 60, 315–327. doi: 10.5334/pb.544 32983549 PMC7500227

[ref17] Hirschi T. Gottfredson M. (2000). In defense of self-control. Theor. Criminol. 4:55. doi: 10.1177/1362480600004001003

[ref18] Hou R. (2009). The influence of emotion on cognitive activities. Psychol. Res. 2, 28–33.

[ref19] Hu Y. Cai Y. Wang R. Gan Y. He N. (2023). The relationship between self-esteem and aggressive behavior among Chinese adolescents: a moderated chain mediation model. Front. Psychol. 14:1191134. doi: 10.3389/fpsyg.2023.1191134, PMID: 37377697 PMC10291261

[ref21] Jiang Y. Tong L. Cao W. Wang H. (2024). Dark triad and relational aggression: the mediating role of relative deprivation and hostile attribution bias. Front. Psychol. 15:1487970. doi: 10.3389/fpsyg.2024.1487970, PMID: 39679158 PMC11641119

[ref22] Kappes A. Schikowski A. (2013). Implicit theories of emotion shape regulation of negative affect. Cognit. Emot. 27, 952–960. doi: 10.1080/02699931.2012.753415 23282147

[ref23] Kirst S. Bögl K. Gross V. L. Diehm R. Poustka L. Dziobek I. (2022). Subtypes of aggressive behavior in children with autism in the context of emotion recognition, hostile attribution Bias, and dysfunctional emotion regulation. J. Autism Dev. Disord. 52, 5367–5382. doi: 10.1007/s10803-021-05387-w 34931277 PMC9637050

[ref24] Laloyaux J. De Keyser F. Pinchard A. Della Libera C. Larøi F. (2019). Testing a model of auditory hallucinations: the role of negative emotions and cognitive resources. Cogn. Neuropsychiatry 24, 256–274. doi: 10.1080/13546805.2019.1629895 31188062

[ref25] Lawler S. Barrett E. L. Teesson M. Kelly E. Champion K. E. Debenham J.. (2024). The long-term effectiveness of a personality-targeted substance use prevention program on aggression from adolescence to early adulthood. Psychol. Med. 54, 2917–2925. doi: 10.1017/S0033291724000989, PMID: 38680095

[ref26] Lin S. Longobardi C. Gastaldi F. G. M. Fabris M. A. (2024). Social media addiction and aggressive behaviors in early adolescents: the mediating role of nighttime social media use and sleep quality. J. Early Adolesc. 44, 41–58. doi: 10.1177/02724316231160142

[ref27] Liu N. Wang J. Yang L. (2022). The impact of social rejection on reactive aggression: the chain mediation of hostile attribution bias and trait anger. Chin. J. Clin. Psychol. 30, 1446–1449. doi: 10.16128/j.cnki.1005-3611.2022.06.037

[ref28] Lyu Y. Su Z. Neumann D. Meidenbauer K. L. Leong Y. C. (2024). Hostile attribution bias shapes neural synchrony in the left ventromedial prefrontal cortex during ambiguous social narratives. J. Neurosci. 44. doi: 10.1523/JNEUROSCI.1252-23.2024 PMC10904091 38316561

[ref29] Ma X. T. (2024). “Noble children” in “poor” vocational schools: a perspective and reflection on academic success of secondary vocational students [in Chinese]. China Vocational Tech. Educ. 16, 69–78.

[ref30] Narzuki I. Basuki A.. (2022). Relationship between self-control and aggressiveness. Proceedings of the Annual Conference on Research, Educational Implementation, Social Studies and History, 91–96.

[ref31] Ong D. C. Goodman N. D. Zaki J. (2018). Happier than thou? A self-enhancement bias in emotion attribution. Emotion 18, 116–126. doi: 10.1037/emo0000309, PMID: 28406680

[ref32] Riemann B. C. Kuckertz J. M. Rozenman M. Weersing V. R. Amir N. (2013). Augmentation of youth cognitive behavioral and pharmacological interventions with attention modification: a preliminary investigation. Depress. Anxiety 30, 822–828. doi: 10.1002/da.22127, PMID: 23658147 PMC4005412

[ref9001] Salavera C. Usán P. Jarie L. (2017). Emotional intelligence and social skills on self-efficacy in Secondary Education students. Are there gender differences? Journal of Adolescence, 60, 39–46. 28750267 10.1016/j.adolescence.2017.07.009

[ref33] Shachar K. Ronen-Rosenbaum T. Rosenbaum M. Orkibi H. Hamama L. (2016). Reducing child aggression through sports intervention: the role of self-control skills and emotions. Child Youth Serv. Rev. 71, 241–249. doi: 10.1016/j.childyouth.2016.11.012

[ref34] Shi G. C. Zhang L. H. Fan H. Y. (2017). A meta-analysis of the relationship between aggression and self-esteem [in Chinese]. Adv. Psychol. Sci. 25, 1274–1288.

[ref35] Song X. F. Cao Y. M. Lin X. N. Ji L. Q. Zhang W. X. (2025). HPA axis genes, peer victimization, and adolescent aggressive behavior: the mediating role of empathy [in Chinese]. Psychol. Dev. Educ. 41, 550–560. doi: 10.16187/j.cnki.issn1001-4918.2025.04.11

[ref36] Subra B. (2023). Why narcissists are more likely to be aggressive? The role of hostile attribution bias. Int. J. Psychol. 58, 518–525. doi: 10.1002/ijop.12924 37286231

[ref37] Tan S. Guo Y. (2008a). Revision of the self-control scale for college students. Chin. J. Clin. Psych. 5, 468–470.

[ref38] Tan S. Guo Y. (2008b). Theoretical assumptions and related research on limited self-control. Chin. J. Clin. Psych. 3, 309–311.

[ref39] Tice D. M. Baumeister R. F. Shmueli D. Muraven M. (2007). Restoring the self: positive affect helps improve self-regulation following ego depletion. J. Exp. Soc. Psychol. 43, 379–384. doi: 10.1016/j.jesp.2006.05.007

[ref40] Van Bockstaele B. van der Molen M. J. van Nieuwenhuijzen M. Salemink E. (2020). Modification of hostile attribution bias reduces self-reported reactive aggressive behavior in adolescents. J. Exp. Child Psychol. 194:104811. doi: 10.1016/j.jecp.2020.104811 32093878

[ref41] Wu C. He G. (2011). Emotional factors in self-control behavior. Psychol. Sci. 34, 317–321. doi: 10.16719/j.cnki.1671-6981.2011.02.026

[ref42] Xiao Y. S. (2021). A study on problem behavior and intervention strategies of secondary vocational school students [in Chinese]. Adv. Soc. Sci. 10, 2697–2704. doi: 10.12677/ass.2021.109370

[ref43] Xu T. Li H. Yan Z. Zhang G. (2024). The effects of self-control on bullying behaviour among martial arts practicing adolescents: based on the analyses of multiple mediation effects. Int. J. Sport Exerc. Psychol. 22, 92–105. doi: 10.1080/1612197X.2022.2116470

[ref9002] Xu X. Wu Y. Xu Y. Ding M. Zhou S. Long S. (2023). The Role of Parent-Child Attachment, Hostile Attribution Bias in Aggression: A Meta-Analytic Review. Trauma, Violence & Abuse. 10.1177/15248380231210920 37970850

[ref44] Xu X. Wu Y. Xu Y. Ding M. Zhou S. Long S. (2024). The role of parent–child attachment, hostile attribution bias in aggression: a meta-analytic review. Trauma Violence Abuse 25, 2334–2347. doi: 10.1177/15248380231210920, PMID: 37970850

[ref46] Yue J. F. Wang S. D. (2025). China vocational education development report (2014–2023) [in Chinese]. Vocational Tech. Educ. 46, 72–79.

[ref45] Yu G. L. Zhang Z. (2021). Issues, characteristics, and countermeasures of mental health education in secondary vocational schools [in Chinese]. China Vocational Tech. Educ. 31, 12–17.

[ref9003] Yu S. Zhang C. Y. Xu W. (2022). Longitudinal Relationships Between Trait Mindfulness and Anxiety and Aggression Among College Students: The Mediating Role of Psychological Resilience and the Moderating Role of Left-Behind Experience. Psychological Development and Education, 38, 711–719.

[ref47] Zhao X.-Y. Zheng S.-J. (2024). The effect of peer victimization on adolescents’ revenge: the roles of hostility attribution bias and rumination tendency. Front. Psychol. 14:1255880. doi: 10.3389/fpsyg.2023.1255880, PMID: 38282847 PMC10812118

